# Population-Related Variability in Qualitative and Quantitative Secondary Metabolite Profile of *Gentianella austriaca* (A. & J. Kern.) Holub

**DOI:** 10.3390/plants12132434

**Published:** 2023-06-23

**Authors:** Zorica Popović, Vera Vidaković, Tatjana Mijalković, Dijana Krstić-Milošević

**Affiliations:** 1 Department of Ecology, Institute for Biological Research “Siniša Stanković”—National Institute of the Republic of Serbia, University of Belgrade, Bulevar Despota Stefana 142, 11060 Belgrade, Serbia; vera.vidakovic@ibiss.bg.ac.rs; 2 Department of Plant Physiology, Institute for Biological Research “Siniša Stanković”—National Institute of the Republic of Serbia, University of Belgrade, Bulevar Despota Stefana 142, 11060 Belgrade, Serbia; mijalkovic.t24@gmail.com (T.M.); dijana@ibiss.bg.ac.rs (D.K.-M.)

**Keywords:** secoiridoids, flavone-*C*-glucosides, xanthones, intraspecies and intrapopulation variability

## Abstract

Phytochemical profiling of six natural populations of *Gentianella austriaca* was performed by HPLC identification and quantification of a number of secondary metabolites, and evaluation of time series of peak areas by chemometric analysis. Phytochemical analysis of *G. austriaca* revealed the presence of iridoids, flavone-*C*-glucosides and xanthones. Twelve secondary metabolites were identified in the aerial parts, roots and seeds, including swertiamarin (SWM), gentiopicrin (GP), sweroside (SWZ), isoorientin (ISOOR), swertisin (SWE), demethylbellidifolin-8-*O*-glucoside (DMB-8-*O*-glc), bellidifolin-8-*O*-glucoside (BDF-8-*O*-glc), mangiferin (MGF), corymbiferin (CBF), corymbiferin-1-*O*-glucoside (CBF-1-*O*-glc), bellidifolin (BDF) and campestroside. Multivariate statistical analyses showed relatively low variability among populations according to secondary metabolite content. However, some pharmacologically important compounds were found in higher amounts in a few populations, which could be useful for conservation and future biotechnological procedures.

## 1. Introduction

The biennial gentian *Gentianella austriaca* (A. & J. Kern.) Holub is an endemic species that inhabits mountainous semi-dry grasslands, pastures and wet meadows. The species usually forms large populations and metapopulations in the mountains, while its populations in the lowlands are smaller and more isolated [1]. The loss of its natural habitats, particularly oligotrophic meadows, due to land-use changes makes this species highly endangered in Central Europe [2]. It is found in the central mountains of Serbia at altitudes above 2000 m, but also on the plateaus at medium altitudes at 1000 m a.s.l. [3].

Gentianellas, have long been used in traditional medicine of South America as substitutes of related Gentiana species, to treat digestive problems [4]. The aqueous extract of *Gentianella nitida* whole plant is used in traditional Peruvian medicine as a remedy for hepatitis, as a cholagogue, and in the treatment of obesity [5]. Recently, Leskovac et al. have reported that the aqueous-ethanolic extract of aerial parts of *G. austriaca* displays radioprotective effect on human lymphocytes in vitro [6]. All gentians were characterized by the presence of three classes of secondary metabolites typical of Gentianaceae family, such as secoiridoids, flavonoids, and xanthones. Earlier phytochemical studies of the genus *Gentianella* resulted in the isolation of xanthone aglycones and glycosides, *C*-glucoflavones, iridoids [7,8,9,10], esterterpenoids [11,12,13], ursolic and oleanolic acids [9]. Bitter principles (secoiridoids), which are prevalent constituents of the genera *Gentiana*, *Gentianella*, *Swertia*, and *Centaurium*, stimulate gastric juice and bile secretion, improving appetite and digestion. Furthermore, they have beneficial pharmacological effects on the central nervous system and smooth muscle relaxation, whereas flavonoids have antidepressant, antiglioma, immunostimulant, antiplatelet aggregation, hypoglycemic, and antihyperlipidemic properties [14]. *C*-glucoflavones are widely distributed in monocots, but only in a few dicot families, such as Leguminosae, Gentianaceae, and Asteraceae [15]. The most common *C*-glucoflavones in *Gentiana* species are isovitexin and isoorientin, both of which are biosynthetically primary compounds. The *Swertia* genus produces mostly isovitexin and swertisin, whereas *Gentianella* species contain isoorientin and swertisin [16].

Naturally occurring xanthones with 1,3,5,8-, and less frequently 1,3,7,8-oxidation pattern are typical for gentianellas. These xanthones belong to the bellidifolin type and mostly appear in the form of *O*-glycosides. Along with them, some *Gentianella* species contained xanthones with additional oxygenation at C-4. Hence, the chemosystematically oriented study of four *Gentianella* species from Balkan Peninsula revealed existence of 1,3,4,7,8-oxygenated xanthone for the first time in European gentianellas [17]. This runs contrary to the study previously published by Carbonier et al. (1977) [18] who have reported that this oxygenated pattern, alongside 1,3,4,5,8-, is uniquely found in New Zealand representatives. In the same study, the aerial parts of *G. austriaca* and *G. bulgarica* were reported to contain campestroside [17], the compound previously found in *G. campestris* [19], *G. germanica*, and *G. ramose* [7]. Campestroside was the distinguishing compound in the phytochemical profile of the roots of three *Gentianella* species (detected in *G. austriaca* and *G. bulgarica*, but not in *G. albanica*) [20].

In vitro propagation of *G. austraica* shoot cultures for the production of xanthones has been successfully developed [21], and in vitro studies have confirmed the pharmacological effects of *G. austriaca* extracts [22]. Studies suggest that naturally occurring xanthones exhibit a variety of in vitro and in vivo pharmacological activities which include hypoglycemic, antitumor, antioxidant, antihepatotoxic, CNS depressant, or stimulant effects [23]. Evaluation of the structure-activity relationship in a series of structurally related xanthones from *G. austriaca* and *G. kochiana* disclosed that dihydroxylation at positions 7, 8 of the xanthone nucleus is the key structural feature responsible for the ability to induce microtubule-associated G2/Mcell block and apoptotic cell death in glioma cells [24]. Two tetraoxidenated xanthones with potent MAO A inhibitory activity were found in *G. austriaca* [17]. Although *G. austraica* and its xanthones have been shown to have neuropharmacological in vitro activity, further research on its antidepressant potential based on in vivo studies is needed [22].

Plant secondary metabolites are well-established as important components in both biochemical and ecological processes, given their role in plant survival and successful reproduction in natural habitats [25]. Considering the exposure to constantly changing biotic and abiotic environments, continuous adjustment of plant morpho-physiological traits, including secondary metabolite profile is required [26,27]. In this regard, the presence and amount of certain phytoconstituents vary widely within species and these variations are related to plant ontogeny, environmental, and genetic factors, and are believed to reflect species’ ability to adapt and evolve rapidly [28]. The chemical diversity of plant populations can be assessed by holistic qualitative and quantitative analysis of all metabolites present within a biological system under specific conditions (metabolomics approach), or by quantitative and qualitative analysis of target compounds (traditional approach), which can provide the insight into variation in chemical phenotypes of species [29].

Intraspecific variability of secondary metabolites has become a hot topic in a new research field called “eco-metabolomics”, which links metabolomics with ecology to understand the biochemical mechanisms governing species interactions with the environment and with other mechanisms [30]. More empirical data on intraspecific variability of secondary metabolites are needed for quality control of active phytochemicals, which is currently one of the main concerns within the area of medicinal plant use [31]. This study is aimed to (a) qualitatively and quantitatively determine the targeted secondary metabolites and (b) evaluate intraspecific variability of secondary metabolites in natural populations of *Gentianella austriaca*.

## 2. Results

**Figure 1:**
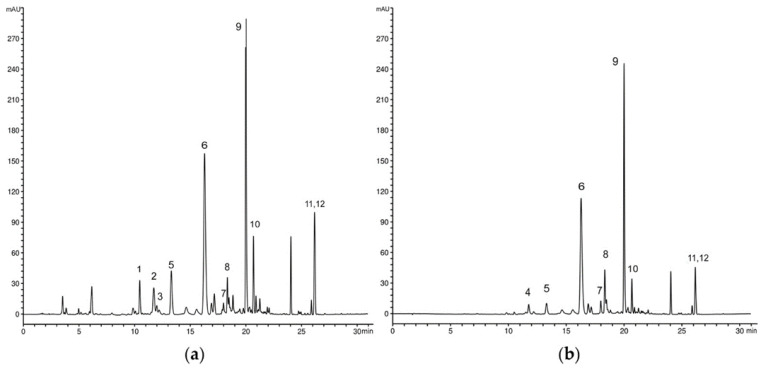
Representative HPLC profile of methanol extract of *G. austriaca* aerial parts recorded at (**a**) λ = 260 nm and (**b**) λ = 320 nm. Peaks: 1—swertiamarin, 2—gentiopicrin, 3—swerodside, 4—mangiferin, 5—campestroside, 6—demethylbellidifolin-8-*O*-glucoside, 7—isoorientin, 8—swertisin, 9—bellidifolin-8-*O*-glucoside, 10—corymbiferin-1-*O*-glucoside, 11—bellidifolin, 12—corymbiferin.

**Figure 2:**
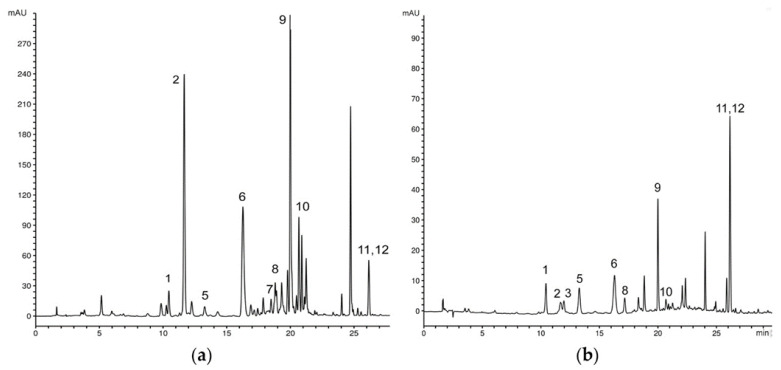
Representative HPLC profiles of methanol extract of *G. austriaca* (**a**) roots and (**b**) seeds recorded at λ = 260 nm. Peaks: 1—swertiamarin, 2—gentiopicrin, 3—swerodside, 4—mangiferin, 5—campestroside, 6—demethylbellidifolin-8-*O*-glucoside, 7—isoorientin, 8—swertisin, 9—bellidifolin-8-*O*-glucoside, 10—corymbiferin-1-*O*-glucoside, 11—bellidifolin, 12—corymbiferin.

Chromatographic analysis of the methanol extracts of *G. austriaca* natural populations indicated that all tested samples have a similar profile of secondary metabolites (Figure 1 and Figure 2).

Three secoiridoids were identified including swertiamarin (1), gentiopicrin (2) and swerodside (3), as well as two *C*-glucoflavones isoorientin (7) and swertisin (8), whose permanent coexistence is specific for *Genianella* species. The xanthone composition consists of seven identified compounds. Among them, three xanthones occure in the form of glucosides-bellidifolin-8-*O*-glucoside (9), demethylbellidifolin-8-*O*-glucoside (6) and corymbiferin-1-*O*-glucoside (10). In addition to glucosides, corresponding aglycones bellidifolin (11) and corymbiferin (12) have also been identified. A very specific compound of gentianellas, campestroside (5), which is a partially saturated analogue of the demethylbellidifolin-8-*O*-glucoside, has been detected in all the samples. This xanthone was tentatively identified based on comparison of UV spectral data with reference data obtained from our previous studies on tetra-substituted xanthones [17]. Mangiferin (4), xanthone *C*-glucoside, was detected in a smallest quantity among the xanthones.

**Figure 3:**
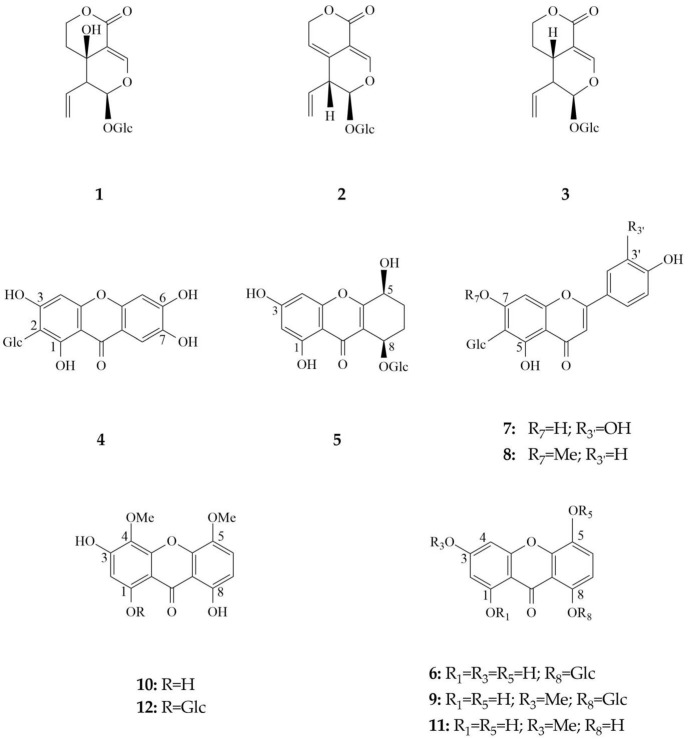
Chemical structures of the secondary metabolites identified in *G. austriaca* plants. 1—swertiamarin, 2—gentiopicrin, 3—swerodside, 4—mangiferin, 5—campestroside, 6—demethylbellidifolin-8-*O*-glucoside, 7—isoorientin, 8—swertisin, 9—bellidifolin-8-*O*-glucoside, 10—corymbiferin-1-*O*-glucoside, 11—bellidifolin, 12—corymbiferin.

Chemical structures of the secondary metabolites identified in *G. austriaca* plants were shown in Figure 3.

**Figure 4:**
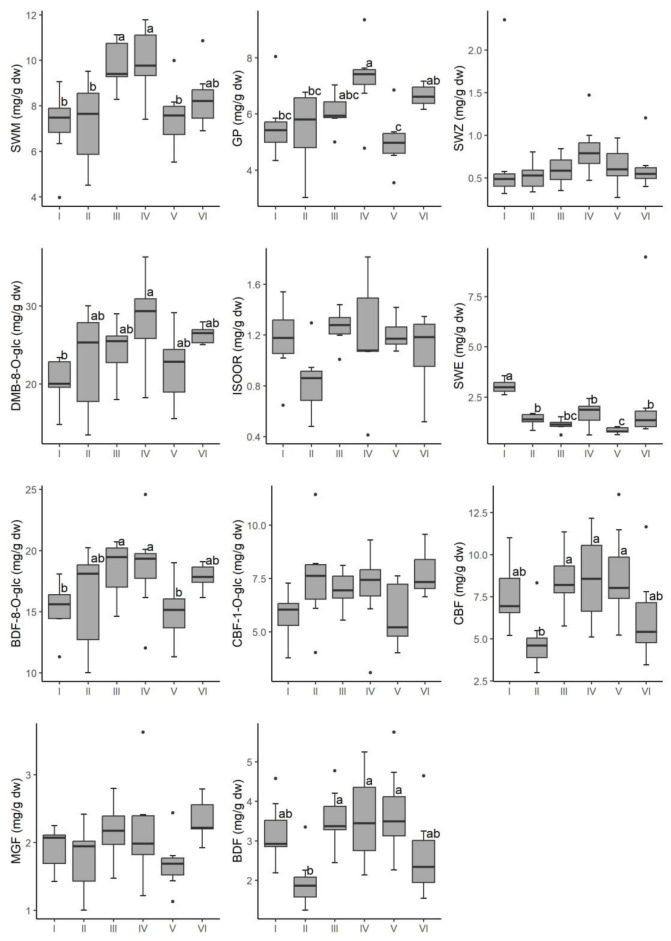
Inter-population differences in the content of 11 secondary metabolites of six *G. austriaca* populations based on ANOVA and Kruskal-Wallis test. Different letters (a, b, c) indicate statistically significant differences, and grouped letters (ab, abc, bc) indicate the lack of significant differences between grouped variables.

Figure 4 visualizes the results of parametric and non-parametric tests performed on the contents of eleven secondary metabolites in aboveground plant parts of six populations. The contents of seven secondary metabolites (SWM, GP, DMB-8-*O*-glc, SWE, BDF-8-*O*-glc, CBF, and BDF) differed significantly between populations, whereas the contents of four secondary metabolites (SWZ, ISOOR, CBF-1-O-glc, and MGF) did not. Population IV had the highest levels of SWM, GP, DMB-8-*O*-glc, CBF, and BF, as well as very high levels of SWM and BDF-8-*O*-glc. Furthermore, populations III and V had significantly high levels of certain secondary metabolites (Figure 4).

**Figure 5:**
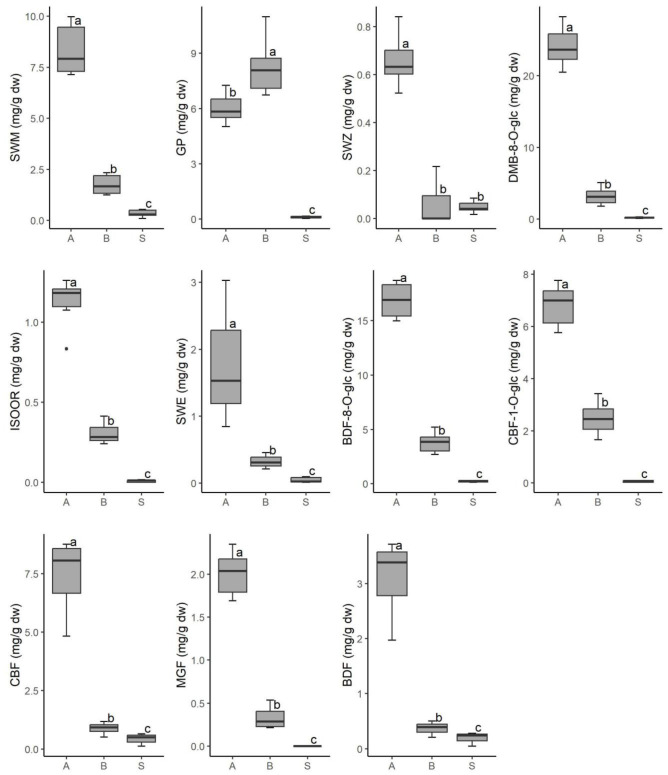
The contents of 11 secondary metabolites in aboveground plant parts (A), belowground plant parts (B) and seed (S) in *G. austriaca*. Different letters (a, b, c) indicate statistically significant differences.

Regarding the plant part, the highest contents of the investigated secondary metabolites were found in the aboveground parts (A), followed by the belowground parts (B), and the lowest contents were present in the seeds (S), except for GP (B > A > S) and SWZ (A > B = S) (Figure 5).

**Figure 6:**
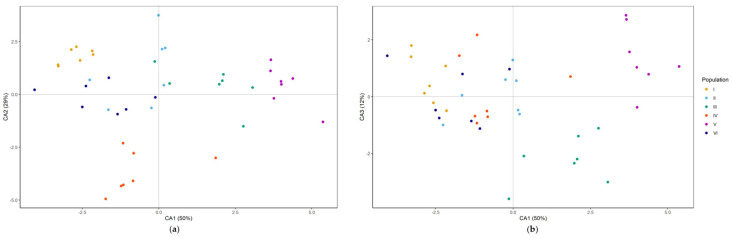
Discriminant analysis of the secondary metabolites from six *G. austriaca* populations. Positions of the analyzed individuals and populations within the first and second (**a**), and first and third (**b**) discriminant axes.

Linear discriminant analysis (LDA) was performed on the set of six groups of individuals belonging to six populations. The first discriminant function explained 50%, the second 29%, and the third 12% of the discrimination (Table 1). The first discriminant function was mainly determined by the contents of BDF and CBF, the second by the contents of CBF and GP, and the third by the contents of DMB-8-*O*-glc and BDF-8-*O*-glc (Table 1). The first discriminant function mainly distinguished Population V, the second Population IV, and the third Population III, from the other populations (Table 2 and Figure 6). DA showed clear separation of Populations I, III, IV and V, and the overlap of Populations II and VI (Figure 6a,b).

## 3. Discussion

Medicinal plants from the gentian family (Gentianaceae) are highly valued as sources of pharmacologically important compounds. However, many of them are facing declining populations due to climate change and habitat loss [32]. Considering that the majority of species in this family are endemic and have low germination capacity, uncontrolled collection for traditional use and overexploitation for commercial purposes pose additional threats. The first in situ conservation measure for these species should be the preservation of natural populations and habitats, followed by ex situ measures such as biotechnology based on plant tissue culture methods. Phytochemical profiling of natural populations is an important step in both conservation approaches, especially when using in vitro methods because the content of some natural compounds has been shown to vary with habitat [33].

Studies on chemodiversity among plant populations have been commonly conducted in the context of natural genetic variation and are based on distance-based measurements [34,35,36,37] or pronounced differences in environmental conditions [38,39]. Populations from non-distant sites with similar environmental patterns, however, offer a more promising opportunity for better understanding naturally occurring variability in species phytochemical profiles. Evidence suggests that intraspecific variation of phytochemicals can exist at a few meters’ scale, and even when individuals have the same secondary chemistry composition, the amounts can be significantly different [40]. This can be attributed to resource heterogeneity, genetic differences, and herbivore or pathogen impact [41]. Considering that plant chemical properties can change in damaged tissues, population/individual life histories can also contribute to this variability [42]. Given that chemically mediated plant-plant interactions are mostly based on secondary metabolite amounts, some authors suggest that intraspecies variability in this trait should be taken into account in allelopathic community research [43]. High interpopulation diversity among natural populations indicates functional redundancy and versatility at the species level [44]. Low interpopulation chemodiversity, on the other hand, may be the result of lower genetic variability and genetic inbreeding [45]. Habitat fragmentation and isolation of small populations has a negative impact on population viability due to a lack of genetic variation, which is required for ecological plasticity [46]. Furthermore, small and isolated populations are often faced with pollen limitation [47], and their fitness can be linked to the presence of jointly flowering species that can either attract [48] or compete for pollinators [49]. Pollen limitation, genetic deterioration, population size decline, and clonal spreading pose significant challenges for endemic species, which are typically found in restricted areas [50]. *G. austriaca* populations on Maljen Mt. had less than 40 reproductive (flowering) individuals spread out in small patches, making it difficult to attract enough pollinators. Multivariate analyses of the *G. austriaca* dataset revealed inconspicuous separation and partial overlap of individuals, indicating relatively low interpopulation variability based on the secondary metabolites investigated. Interpopulation variability of secondary metabolites was found to be similarly low in small populations of *Gentiana pneumonanthe* [51] and *Gentiana asclepiadea* [52].

The greatest differences in secondary metabolite content were found between plant parts. The majority of the compounds tested were found in both below- and aboveground plant parts and seeds. Only GP was found in comparable amounts in both below- and aboveground parts, whereas the amounts of other compounds were much lower in belowground parts. SWZ (not found in belowground parts, or only in traces in two populations) and MFG and ISOOR (not found in seeds, or only in traces) displayed the most prominent differences between the content of secondary metabolites in plant parts. Secondary metabolite biosynthesis and accumulation demonstrate organ and/or tissue specificity, primarily in four plant morphological compartments: roots and stems, leaves, flowers, fruits, and seeds [53]. These processes are influenced by genetic, environmental, and developmental factors, all of which have a synergistic effect on secondary metabolite biosynthesis and accumulation and may show large-scale range depending on overall photosynthetic and energy metabolism [54]. Secondary metabolite accumulation in roots and stems, for example, is primarily influenced by growth periods, growth seasons, and growth years [52,55]; leaves, with their primarily photosynthetic function, can also store secondary metabolites, which vary with leaf age and growth stage [56,57]; variations in secondary metabolites synthesis and accumulation in flowers are strongly influenced by developmental stages and circadian rhythm [58]; seeds also show developmental stage variations, which are related to gene expression, i.e., enzymes responsible for secondary metabolites biosynthesis [59]. Secondary metabolites are important in seed dispersal and fruit defense [60], and there is evidence that reproductive tissues and organs (flowers, fruits, seeds) have distinct secondary metabolite profiles and higher amounts of secondary metabolites [61]. The qualitative-quantitative analysis of *G. austrica* in late flowering/early fruiting stage reveals a similar set of secondary metabolites in all plant parts.

The findings show that the content of four compounds (SWZ, ISOOR, CBF-1-*O*-glc, and MGF) was similar among all populations studied. However, the amounts of seven compounds differed significantly between populations, contributing to the separation of populations III, IV and V based on the high contents of BDF, DMB-8-*O*-glc, BDF-8-*O*-glc CBF, and GP. This result suggests the importance of using qualitative/quantitative analyses in phytochemical profiling of species. Quantification of compounds of pharamacological interest can provide information about intraspecies chemodiversity, which is the fundamental for bioprospecting, and can lead to multidisciplinary approaches encompassing ecological, biological, chemical, pharmaceutical, and biomedical disciplines [62]. The majority of gentianelas’ xanthones are bellidifolins. They are commonly found as *O*-glycosides and are considered to be carriers of a variety of pharmaceutical properties. The two dominant chromatogram peaks belong to demethylbellidifolin-8-*O*-glucoside and bellidifolin-8-*O*-glucoside, xanthones with 1,3,5,8-oxidation pattern characteristic for *Gentianella* species. Bellidifolin and demethylbellidifolin are both antioxidants with antimicrobial, anti-diabetic, and cardioprotective properties, with inhibitory effects on acetylcholinesterase and monoamino oxidase [16], and bellidifolin also has hypoglycemic and neuroprotective properties [63,64]. Bellidifollin reduces oxidative stress and shows hepatoprotective capacity [65], and inhibit acetylcholinesterase (AChE) activity [66]. Evidence of a broad range of pharmacological activities encouraged efforts toward in vitro propagation of gentinellas, as well as testing the conditions for optimal xanthone production in shoot cultures [67]. However, due to the scarcity of plant material (rare, endemic species that grow in inaccessible locations), data on the phytochemistry of these species, particularly the variability within natural populations, is limited. More data on this topic can aid in mapping the locations and populations with high concentrations of secondary metabolites of interest, which can be used as a starting point for future biotechnological research.

## 4. Materials and Methods

### 4.1. Study Site

**Figure 7:**
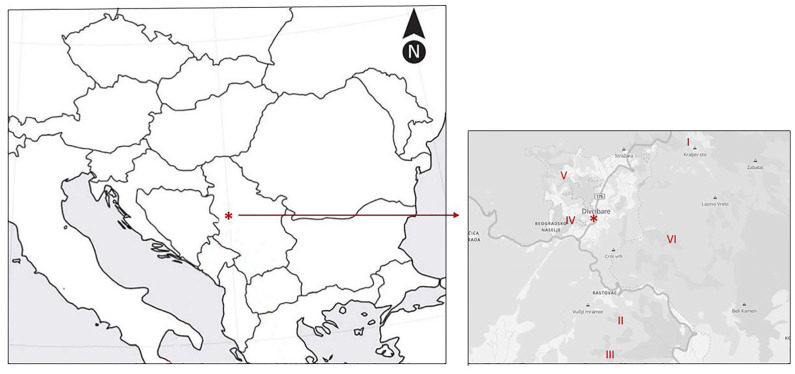
A map of investigated area. An asterisk (*) denotes the central location (Divčibare) on the larger map (position of Serbia on the Balkan Peninsula) and the smaller map (investigated area). *G. austriaca* populations are denoted by numbers (I–VI).

Field research was conducted at Divčibare (44°06′13″ N, 19°59′19″ E), which is located in the central part of Mt. Maljen in northwest Serbia. The six *G. austriaca* populations studied were spread over a 5 km × 5 km area (Figure 7), distributed in small patches in meadow communities, and accounted for approximately 30–40 mature individuals (generative adults with flowering stalks). Table 3 shows the coordinates and altitudes of the studied populations.

### 4.2. Plant Material and Sample Preparation

The field research was carried out in August 2022. Seven individuals from each population were harvested immediately following flowering (late flowering/early fruiting phase). The collected plants were determined by Dr. Z. Popović at the Institute for Biological Research, Department of Ecology, using standard keys for the determination of the plant species [3,68]. Plants were and air-dried in the laboratory. Each plant was divided into two parts: belowground and aboveground. The flowers and fruits were separated from the stem and seeds that fell out of the dried fruits were collected. Further analyses were performed on the vegetative aboveground parts (A) (7 individuals × 6 populations, *n* = 42). Belowground parts (B) and seeds (S) were analyzed as a mixed sample per population due to the small amount of material (thin roots and small amount of seeds).

### 4.3. Analytical Procedure

Samples including belowground and aboveground plant parts, and seeds, were ground to fine powder using a mortar and a pestle. Fifty milligrams of powdered samples were extracted with 1 mL of methanol (HPLC grade, J.T. Baker, Deventer, The Netherlands) in an ultrasonic bath at room temperature for 20 min. After sonication extracts were centrifuged at 10,000 rpm for 10 min. The supernatants were filtered through 0.45 μm nylon syringe filters (Captiva Econo Filters, 13 mm, Agilent Technologies, Waldbronn, Germany) before injection into the HPLC system for analysis.

Identification and quantification of secondary metabolites in tested extracts were carried out on an Agilent Series 1100 HPLC system equipped with diode array detector (DAD). A reverse phase ZorbaxSB-C18 (Agilent) analytical column (150 × 4.6 mm, 5 μm) was used for the component separation. The mobile phase consisted of solvent A (1%, *v*/*v* solution of orthophosphoric acid in water) and solvent B (acetonitrile, HPLC grade, J.T. Baker, Deventer, The Netherlands) using a gradient elution of 98–90% A 0–3 min, 90% A 3–6 min, 90–85% A 6–8 min, 85% A 8–13 min, 85–70% A 13–18 min, 70–30% A 18–25 min, and 30–0% A 25–30 min. Column elution was monitored at 260 and 320 nm, with a flow rate of 1 mL min^−1^.

Standards of xanthone glucosides demethylbellidifolin-8-*O*-glucoside, bellidifolin-8-*O*-glucoside and corymbiferin-1-*O*-glucoside, aglycones corymbiferin and bellidifolin, as well as *C*-glucoflavones swertisin and isoorientin were isolated previously in our laboratory from the aerial parts of *Gentianella albanica* [17]. Camprestroside was isolated from aerial parts of *G. austriaca* [17]. Mangiferin was purchased from Sigma-Aldrich Inc., Germany/USA. Secoiridoids swertiamarin, gentiopicrin and sweroside were bought from Cfm Oscar Tropitzsch (Bayern, Germany).

Stock solutions of standards were prepared by dissolving compounds in methanol.

The quantification of secondary metabolites was done using the external standard method by preparing calibration standards and recording the calibration curves at 260 nm for secoiridoids, *C*-glucoflavones and xanthones, and at 320 nm for xanthones mangiferin and bellidifolin. The results are presented as mg/g of dry weight (dw).

### 4.4. Statistical Analysis

Six populations of *G. austriaca* were compared on the basis of the contents of eleven secondary metabolites (secoiridoids, flavonoids and xanthones). The assumption of normality was tested with the Shapiro-Wilk test (*p* > 0.05 indicated normal distribution). ANOVA was applied for comparing the populations when the variables appeared to follow normal distribution, whereas a non-parametric Kruskal-Wallis rank sum test was used when the variables did not appear to follow a normal distribution. The False Discovery Rate (FDR) *p*-adjustment method was used to control the type I error rate in hypothesis testing. Linear discriminant analysis (LDA) was carried out on log-transformed data (y′ = log10(y + 1), y = original data value) in order to visualize the data structure. Statistical analyses were performed using R statistical software, version 4.2.2 [69].

## 5. Conclusions

The findings suggest that *G. austriaca* is a rich source of pharmacologically important compounds. The overall intraspecies variability in the total dataset was relatively low, which is characteristic of small and isolated populations. However, the amounts of some compounds (DMB-8-*O*-glc, BDF-8-*O*-glc) with high pharmacological potential (inhibitors of monoamine oxidases A, cardiovascular protective and antitumor effect) were significantly higher in a few populations, which could be useful for conservation and future biotechnological procedures. These findings highlight the significance of qualitative/quantitative analyses of natural populations and evaluating their quality-related characteristics.

## Figures and Tables

**Table 1:** Standardized coefficients for the first three canonical axes (CA) of variation in *G. austriaca* secondary metabolites from the discriminant functional analysis of six a priori defined groups. The variables that contribute the most to distinguishing the populations are bolded.

Compound	CA1	CA2	CA3
SWM	−0.222	−1.364	−0.982
GP	−0.977	**−2.918**	−0.352
SWZ	−0.247	−0.506	−0.321
DMB-8-*O*-glc	0.169	−2.037	**3.955**
ISOOR	0.084	−1.038	−0.334
SWE	−1.376	0.390	0.475
BDF-8-*O*-glc	1.105	2.216	**−3.160**
CBF-1-*O*-glc	1.650	1.710	0.025
CBF	**−15.649**	**3.467**	0.141
MGF	−2.029	1.894	−0.441
BDF	**16.863**	−1.903	0.854
Eigenvalue	6.171	3.593	1.429
Explained variation (%)	50.286	29.274	11.643

**Table 2:** Means of canonical variables from the discriminant functional analysis of six a priori defined groups of *G. austriaca*. Distinct mean values are bothed.

Population	CA1	CA2	CA3
I	−2.723	1.814	0.584
II	−0.529	1.124	0.062
III	1.742	0.427	**−2.246**
IV	−0.731	**−3.677**	0.215
V	**4.117**	0.448	1.383
VI	−1.877	−0.137	0.002

**Table 3:** Geographic locations of the studied populations.

Population	Longitude	Latitude	Altitude (m)
I	44°4′12″	20°0′22″	1075
II	44°6′3″	19°59′4″	990
III	44°3′33″	19°35′19″	998
IV	44°4′16″	20°0′5″	1045
V	44°4′19″	20°0′28″	1042
VI	44°4′45″	20°0′31″	995

## Data Availability

The data presented in this study are available from the authors.
